# A single-copy knock-in system: one plasmid to target all chromosomes in *C. elegans*

**DOI:** 10.1093/g3journal/jkaf220

**Published:** 2025-09-19

**Authors:** Erica Dinneen, Purbasha Dasgupta, Avinash Sharma, Khairun Nisaa, Carlos G Silva-García

**Affiliations:** Department of Molecular Biology, Cell Biology, and Biochemistry, Brown University, Providence, RI 02903, United States; Center on the Biology of Aging, Brown University, Providence, RI 02903, United States; Department of Molecular Biology, Cell Biology, and Biochemistry, Brown University, Providence, RI 02903, United States; Center on the Biology of Aging, Brown University, Providence, RI 02903, United States; Department of Molecular Biology, Cell Biology, and Biochemistry, Brown University, Providence, RI 02903, United States; Center on the Biology of Aging, Brown University, Providence, RI 02903, United States; Department of Molecular Biology, Cell Biology, and Biochemistry, Brown University, Providence, RI 02903, United States; Center on the Biology of Aging, Brown University, Providence, RI 02903, United States; Department of Molecular Biology, Cell Biology, and Biochemistry, Brown University, Providence, RI 02903, United States; Center on the Biology of Aging, Brown University, Providence, RI 02903, United States

**Keywords:** *C. elegans* methods, CRISPR/Cas9, genome engineering, single-copy knock-in, transgenesis

## Abstract

Successful transgenesis in model organisms has significantly helped us understand gene function, regulation, genetic networks, and potential applications. Here, we introduce a single-copy knock-in system that uses 1 plasmid to target all chromosomes in *Caenorhabditis elegans* (SKI PLACE), designed for inserting a transgene by CRISPR/Cas9. The SKI PLACE system uses the pSKI plasmid to insert a desired transgene at specific harbor loci on each chromosome. The pSKI plasmid contains multiple restriction sites for cloning and serves as a CRISPR/Cas9-based insertion repair template because it has 2 synthetic and long homology arms that recombine with the SKI PLACE cassettes. This system also uses a single crRNA guide, which acts as a Co-CRISPR enrichment marker. Overall, the SKI PLACE system is flexible; with the same SKI PLACE cassette on each chromosome, researchers can select the insertion site, work with 1 plasmid, and streamline tracking using standard primers.

## Introduction

Diverse methods have been developed to express transgenes in *Caenorhabditis elegans*. Traditionally, exogenous genes have been introduced in multi-copy as extrachromosomal arrays (Mello et al. 1991), gamma/UV integration (Evans 2006), or biolistic transformation (Praitis et al. 2001). They can also be expressed in single-copy, using MosSCI-based integration (Frøkjær-Jensen et al. 2008, 2012). These techniques have certainly facilitated essential discoveries. However, the advent of CRISPR/Cas9 technology (Dickinson et al. 2013; Friedland et al. 2013; Kim et al. 2022) has revolutionized the way to modify the *C. elegans* genome, particularly for single-copy transgene expression.

CRISPR/Cas9 (clustered regularly interspaced short palindromic repeats-associated) enables the precise insertion of transgenes through homology-directed repair (HDR) that can label an endogenous gene at its native locus with a fluorescent protein (Friedland et al. 2013; Dokshin et al. 2018; Farboud et al. 2019; Vicencio et al. 2019; Goudeau et al. 2021) or insert a transgene with its regulatory sequences (Philip et al. 2019; Silva-García et al. 2019; Stevenson et al. 2020; Mouridi et al. 2022). Despite CRISPR/Cas9 being widely used in *C. elegans* research, the insertion of exogenous genes and the selection of appropriate protocols continue to pose a challenge. To contribute to existing single-copy insertion protocols, we have developed a single-copy knock-in system: 1 plasmid to target all chromosomes in *C. elegans* (SKI PLACE).

The SKI PLACE system consists of a collection of strains designed to insert a transgene using a single plasmid as a repair template. The SKI PLACE plasmid, pSKI, contains multiple restriction sites that facilitate the cloning of the gene of interest. This pSKI plasmid serves as a repair template for CRISPR/Cas9-based single-copy knock-in insertion at specific harbor loci on each chromosome. Since each chromosome contains the same cassette, researchers can choose the final location for their gene of interest. Our SKI PLACE system offers a new approach, enabling researchers to design their gene of interest, select chromosomal destinations, and track the genetic insertion by standard primers, thereby providing an additional option for inserting single-copy transgenes in *C. elegans*.

## Material and methods

### Strains and maintenance

Bristol N2 strain was provided by the Caenorhabditis Genetics Center (CGC), which is funded by NIH Office of Research Infrastructure Programs (P40 OD010440). The worms in this experiment were grown on nematode growth medium (NGM) plates. These plates were seeded with the *Escherichia coli* bacterial strain OP50-1. The *C. elegans* used in this study were maintained under standard procedures (Brenner 1974).

### Strains and sequences generated in this work

The strains generated in this study are listed in Table 1 and will be available for distribution through the CGC. All sequence files for the SKI PLACE lines are available in the Supplementary material available at FigShare (https://gsajournals.figshare.com) and at https://www.theSGlab.com/resources. All crRNAs, tracrRNA, and oligonucleotides are listed in Supplementary Tables 1 and 2.

**Table 1.** Strains generated in this study.

Chromosome	Strain^a^	Genotype	Outcrossing
Chr. I	CSG18	*N2, gsgIs2 [synthetic 900 bp HA 1::dpy-10 cRNA site:: synthetic 900 bp HA 2, I:2850968]*	6×
Chr. II	CSG60	*N2, gsgIs3 [synthetic 900 bp HA 1::dpy-10 cRNA site:: synthetic 900 bp HA 2, II:9834540]*	8×
Chr. III	CSG36	*N2, gsgIs5 [synthetic 900 bp HA 1::dpy-10 cRNA site:: synthetic 900 bp HA 2, III:7007779]*	6×
Chr. IV	CSG10	*N2, gsgIs1 [synthetic 900 bp HA 1::dpy-10 cRNA site:: synthetic 900 bp HA 2, IV:5014948]*	6×
Chr. V	CSG76	*N2, gsgIs4 [synthetic 900 bp HA 1::dpy-10 cRNA site:: synthetic 900 bp HA 2, V:8644845]*	8×
Chr. X	CSG53	*N2, gsgIs8 [synthetic 900 bp HA 1::dpy-10 cRNA site:: synthetic 900 bp HA 2, X:798667]*	6×

^a^The receipt strain for all SKI PLACE lines was N2.

### Identification of harbor loci

We utilized previously characterized harbor loci to knock in the SKI PLACE cassette. These sites have been characterized in our previous SKI LODGE system (Silva-García et al. 2019). Based on this, the cassette was inserted at the following positions: Chr. I:2850968, Chr. III:7007779, Chr. IV:5014948, and Chr. V:8644845. For position Chr. II:9834540, we analyzed genomic regions used in the MosSCI system (Frøkjær-Jensen et al. 2014). Since the position of Chr. X:798667 was not previously characterized, we selected the chromosome region based on the region that exhibits less transgene silencing (Frøkjær-Jensen et al. 2016). All SKI PLACE sites were chosen in an intergenic region. The version WS297 of the *C. elegans* genome was used.

### Generation of the pSKI plasmid

The SKI PLACE plasmid consists of 2 synthetic homology arms (HAs), 2 genotyping sequences (Gs1 and Gs2), and a multi-cloning sequencing site (MCS). First, we designed the 2 synthetic HAs. Each HA comprises 900 bp with around 50% GC content and was generated using the random DNA sequence generator (Villesen 2007). After each sequence was generated, they were run for Basic Local Alignment Search Tool (BLAST) in order to guarantee the sequences were not found anywhere else in the *C. elegans* genome, thereby preventing unintended off-targets. A similar approach was followed to generate the synthetic Gs1 and Gs2 sequences. The MCS, which resides between Gs1 and Gs2, contains 47 single cuts. The sequences for the HAs and MCS were ordered from Genewiz as single-stranded DNA templates. Gs1 and Gs2 templates were generated via PCR utilizing primers listed in Supplementary Table 2. The plasmid pPD95_75 (Addgene #1494) served as the backbone for the pSKI plasmid. pPD95_75 was digested with HindIII and ApaI (NEB). The digested plasmid was purified using a Zymo Gel DNA Recovery kit (D4007). The single-stranded DNA templates and backbone were ligated following standard Gibson Assembly protocol (Gibson et al. 2009) and transformed into NEB 5-alpha competent *E. coli* (NEB C2987H). The final pSKI plasmid was sequenced to corroborate the edits and can be obtained from Addgene (plasmid #232484).

### Cas9 purification

Cas9-His-tagged was purified by overexpressing in *E. coli* BL21 Rosetta cells. Nickel-NTA beads were used to pull it down, followed by HPLC purification. Briefly, we inoculated LB culture with SpCas9 containing *E. coli* Rosetta cells and agitated for 3 to 6 h until the culture reached an OD of 0.6 to 0.8. The culture was cooled down on ice water to below 20 °C, and 0.5 mM IPTG was added, followed by incubation overnight in a shaker at 20 °C. The culture was spun, and the bacterial pellet was resuspended in 27.5 ml of 50 mM Tris pH 8, 150 mM NaCl, 10% glycerol, and 2 mM TCEP. Cells were lysed by adding 2 ml 10× FastBreak buffer (Promega V8571) and 5 μl Benzonase, incubated for 15 to 20 min at RT, spun at 38,000 × *g* for 15 min at 4 °C, and the supernatant was collected. Ni-NTA resin was washed in an equilibration buffer (50 mM Tris pH 8, 500 mM NaCl and 10% glycerol). The supernatant was then incubated with 1 ml of this prewashed Ni-NTA resin at 4 °C for 1 h. The sample was poured into a disposable column and washed with wash buffer (20 ml of 50 mM Tris pH 8, 500 mM NaCl and 10% glycerol, 20 mM imidazole, and 2 mM TCEP). The sample was then eluted using elution buffer containing 5 ml 50 mM Tris pH 8, 500 mM NaCl and 10% glycerol, 400 mM imidazole, and 2 mM TCEP. The eluted Cas9 protein was diluted 1- to 2-fold with 1× PBS and loaded onto a 5 ml heparin column equilibrated with 1× PBS. The sample was eluted in a linear salt gradient from 0.1 to 1 M NaCl in 1× PBS and 1 ml fractions were collected. Fractions were analyzed on a gel and pooled all Cas9 containing fractions followed by concentrating them down to 3 ml volume with Amicon Ultra-15 filter (30 kDa cutoff, spun 4,000 × *g* 20 min at 4 °C). We exchanged the buffer for 1× PBS, 10% glycerol, and 2 mM TCEP using a PD-10 column. The insoluble protein was spun down at 4 °C for 15 min and concentrated down to 4/12, a final volume of 500 μl. Purified Cas9 was filter sterilized (0.22 μm), and the final concentration was measured by NanoDrop and stored at −80 °C.

### CRISPR/Cas9 mix and microinjection

All CRISPR edits and insertions required to generate and test the strains were performed using the CRISPR protocol adapted from Paix et al. 2015 (Paix et al. 2015) and Silva-García et al. 2019 (Silva-García et al. 2019). Briefly, homology repair templates were amplified by PCR, using primers that introduced a minimum stretch of 35 bp homology to the cassette at both ends. CRISPR injection mix reagents were added in the following order: 0.375 μl HEPES pH 7.4 (200 mM), 0.25 μl KCl (1 M), 1.25 μl tracrRNA (8 μg/μl), 0.5 μl dpy-10 crRNA (8 μg/μl) or 0.5 μl dpy-5 crRNA (8 μg/μl), 0.5 μl dpy-10 ssODN (1000 ng/μl) or 0.5 μl dpy-5 ssODN (1000 ng/μl), and PCR repair template(s) up to 400 ng/μl final in the mix. Water was added to reach a final volume of 8 μl. Then, 2 μl purified Cas9 (12 μg/μl) was added at the end, mixed by pipetting, spun for 2 min at 13,000 rpm, and incubated at 37 °C for 10 min. Mixes were microinjected into the germline of day 1 adult hermaphrodite worms using standard methods (Evans 2006).

### SKI PLACE cassette construction

All SKI PLACE strains were generated by the abovementioned CRISPR protocol, using at least 35 bp of homology as recombination arms. Two templates were used to generate the lines. The templates were amplified from the pSKI plasmid. One template consisted of HA1 and the other of HA2. The genomic *dpy-10* site was added between the 2 templates: HA1::*dpy-10* site::HA2. Each template, including homology arms for the corresponding genomic region and the overlapping sequence for the *dpy-10* site, with ∼980 bp in length. The primers used to amplify the HA1 and HA2 and the specific crRNAs for each strain can be found in Supplementary Tables 1 and 2. In order to introduce the *dpy-10* site into the strains, we used another identifiable Co-CRISPR target gene, *dpy-5* (Silva-García et al. 2019). All final SKI PLACE strains were verified by sequencing and were outcrossed to N2 at least 6 times to remove the Co-CRISPR marker mutation (*dpy-5*) as well as any additional unwanted off-site editions. Although all SKI PLACE strains were outcrossed, we disclaim that no additional study has been conducted on the potential influences of these sequences on animal physiology beyond the data shown in this work.

### Verification of SKI PLACE strains

To validate the strains, we generated a transgene reporter based on our SKI PLACE system. The following transgene was cloned into the pSKI plasmid: *myo-3p::mCherry:unc-54 3′ UTR*. The transgene was amplified from the pCFJ104 plasmid (Addgene #19328) by PCR. Briefly, the pSKI plasmid was digested with XbaI and KpnI (NEB). Then, 2 PCRs were performed, 1 for amplifying the *myo-3p* and 1 for *mCherry::3′ UTR*. The primers utilized in these PCRs are listed in Supplementary Table 2. These 2 purified PCRs and the digested pSKI plasmid were ligated following standard Gibson Assembly protocol and transformed into NEB 5-alpha competent *E. coli* (NEB). After verification, the final plasmid, pED4, was injected into each strain, following the CRISPR protocol from Silva-García et al. 2019 (Silva-García et al. 2019) (see above CRISPR/Cas9 mix section).

Immediately after injection, individual worms were placed at 20 °C. We usually inject 14 worms, single them out into a plate, and obtain dumpies (*dpy*) or rollers (*rol*) from 11 to 13 plates. Note that we do not track every injected worm for potential jackpot events, as we have not observed these types of events in our hands before. Three to four days after injection, all plates were screened for *dpy* or *rol* animals, and each *dpy/rol* was singled out. Then, all these *dpy/rol* were screened for mCherry. Worms showing *dpy/rol* and mCherry phenotype were lysed and underwent PCR for both homology arms (HA1 and HA2). F1 worms were placed in 5 µl of single worm lysis buffer (30 mM Tris pH 8.0, 8 mM EDTA pH 8, 100 mM NaCl, 0.7% NP-40, 0.7% Tween-20, and 100 µg/ml proteinase K) and lysed for 1 h at 60 °C, followed by incubation at 95 °C to inactivate the proteinase K. We then screened for the CRISPR edit by PCR using Apex Taq RED Master Mix 2.0X (Genesee Scientific; it contains Apex Taq DNA polymerase, an ammonium-based buffer system, Apex ultra-pure dNTPs [>99%], magnesium chloride, and a Red Dye for visualization) as recommended by the manufacturer, using 1 µl of worm lysate as a template. Animals were genotyped utilizing the genotyping SKI primers. A more detailed approach regarding genotyping can be found in our step-by-step guide in Supplementary File 1. Strains generated to verify the SKI PLACE strains are listed in Supplementary Table 4.

### Lifespan assays

Lifespan experiments were performed on standard NGM plates at 20 °C. Plates were seeded with the *E. coli* strain HT115. Worms were synchronized by bleaching gravid adult worms, and then the embryos were placed onto plates once bleaching had been completed. Once the embryos reached their first day of adulthood, 150 worms were picked for each strain. These worms were then separated into 6 plates, each containing 25 animals. Worms were transferred to new plates every other day until reproduction ended. Survival was scored every 1 to 2 d; a worm was deemed dead once there was no response to 3 taps to both the head and tail. Worms that had left the NGM, had eggs hatch inside the adult, and had lost vulval integrity were censored. The analysis of the lifespans was performed using GraphPad Prism.

### Generation time assay

Worms were initially synchronized with an egg lay. Once the worms had reached the mid-L4 stage, 20 worms were singled out onto individual 10 mm NGM plates seeded with OP50-1. After 10 h, plates were checked for embryos that had been laid. This process was repeated every hour until every worm had laid an embryo. Each worm's generation time was calculated based on the initial egg laid.

### Brood size assay

Worms were initially synchronized with an egg lay. Once the worms had reached the mid-L4 stage, 20 worms were singled out onto individual 10 mm NGM plates seeded with OP50-1. Worms were transferred to fresh plates for 6 d or until reproduction had ceased. The plates were kept at 20 °C for 3 d, until the progeny had reached the first day of adulthood. The number of progenies from each parental worm was counted daily, and the total sum of progeny laid was calculated.

### Microscopy

Fluorescence imaging of whole worms was performed using Zeiss Axio Imager 2 MAT Upright Microscope. Worms were anesthetized in 10× diluted 100 mM tetramisole solution prepared in M9 1× buffer and mounted on 2% M9 1× agarose pads on glass slides. Images were collected using a 10 × objective and were processed with Fiji ImageJ v2.16.0. Images for each strain were taken on the first and eighth day of adulthood; this process was repeated for both the first and eighth generations.

### Statistical analysis

All statistical analyses were performed using GraphPad Prism (v9.4.1). Nonparametric tests were performed for brood size and generation time assays due to non-normally distributed data, as confirmed by Shapiro–Wilk tests. Data from at least 2 independent biological replicates (*n* = 15 to 20 worms per strain per replicate) were pooled and analyzed using a Kruskal–Wallis test (α = 0.05), followed by Dunn's post hoc test for planned comparisons between each experimental strain and the N2 wild-type control. Survival analyses were conducted using the Log-rank (Mantel–Cox) test (*n* = 85 to 120 worms per condition). Statistical significance was defined as *P* < 0.05 for all experiments.

### Ethics statement

*C. elegans* is not protected under most animal research legislation.

## Results

We sought to build a system to generate single-copy transgenes in *C. elegans*. The SKI PLACE system consists of 2 parts. First, the pSKI plasmid, in which the gene of interest can be introduced by traditional cloning or Gibson reaction (Gibson et al. 2009), is used as a CRISPR/Cas9 repair template. Second, the SKI PLACE strains carry a cassette with identical sequences (homology arms) that flank the gene of interest in the pSKI plasmid. Each strain has 1 cassette per chromosome (I, II, III, IV, V, and X). The following sections describe the development of the SKI PLACE system, how it can be used, and its advantages and limitations.

### The plasmid

**Fig. 1.**
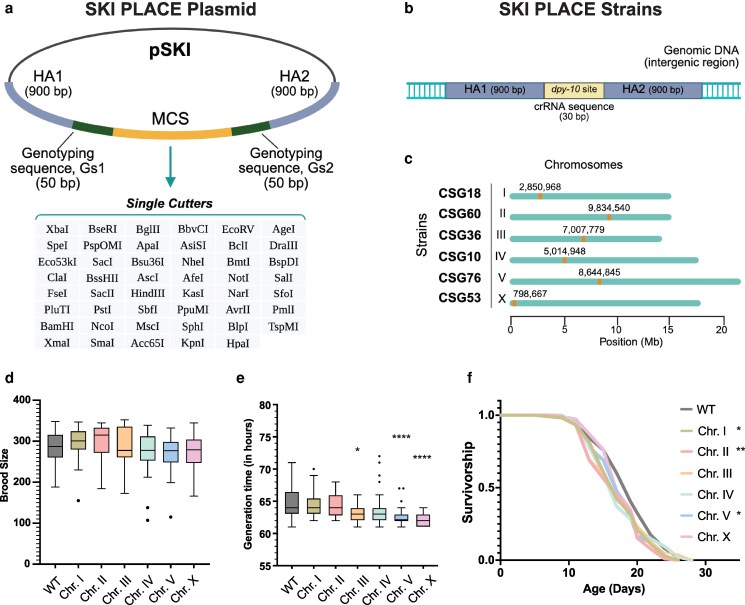
Generation of the SKI PLACE system. a) Schematic depiction of the pSKI plasmid. The pSKI is composed of 2 homology arms (HA1 and HA2, 900 bp each), 2 genotyping sequences (Gs1 and Gs2, 50 bp each), and a multi-cloning site sequence (MCS), which carries 47 single cuts. The table lists the restriction enzymes in 5′ to 3′ order. b) Each SKI PLACE strain carries the *dpy-10* site for the *dpy-10* crRNA, which is flanked by the same HAs that are present in the pSKI plasmid. c) Every SKI cassette was introduced into a defined autosomal location by CRISPR/Cas9. All the outcrossed SKI PLACE lines were tested for brood size d), generation time e), and lifespan f). Panels d) and e) show Tukey box plots summarizing data from ≥2 independent biological replicates. Boxes represent the interquartile range, the line indicates the median, and whiskers extend to 1.5 × IQR. Dots beyond the whiskers denote statistical outliers as defined by the Tukey method. *P* values are based on pairwise comparisons using Dunn's post hoc test following a Kruskal–Wallis test. The f) panel shows 1 replicate of 2; survival curves are compared by the log-rank (Mantel–Cox) method. * < 0.05, ** < 0.01, *** < 0.001, **** < 0.0001. Details about the construction of the SKI PLACE system can be accessed in the Materials and methods section.

To engineer the pSKI plasmid, we first designed a multi-cloning site sequence (MCS) with 47 single cuts (Fig. 1a). To use the pSKI plasmid as a CRISPR template during homologous recombination (Dickinson et al. 2013; Kim and Colaiácovo 2019), we specifically designed 2 synthetic homology arms (HAs). Each HA comprises 900 bp (∼50% GC content) and does not exist in the *C. elegans* genome. We designed the HAs for single-copy insertion by homologous recombination, and the gene of interest (GOI) will reside between them: HA1::GOI::HA2. Since the GOI significantly differs for each single-copy insertion and experiment needs, we added 2 small sequences for genotyping during the PCR screening (genotyping sequences: Gs1 and Gs2). Each Gs has 50 bp, does not exist in the *C. elegans* genome, and flanks the MCS with the final design as HA1::Gs1::MCS::Gs2::HA2 (Fig. 1a). In summary, the researcher can use the MCS region to open the pSKI plasmid and introduce the gene of interest using their preferred cloning method, obtaining the following final design: HA1::Gs1::GOI::Gs2::HA2.

### The strains

Having the pSKI plasmid, we sought to generate transgenic *C. elegans* strains in which a single copy of the HAs has been knocked in at a defined harbor locus. These strains also contain the target sequence of a well-characterized crRNA that could later be used to knock in the GOI by CRISPR/Cas9 and simultaneously serve as a co-CRISPR marker (Fig. 1b).

To define harbor loci for the SKI PLACE system, we used those that we have previously characterized. We developed a single-copy knock-in loci for defined gene expression (SKI LODGE) system that allows rapid single-copy tissue-specific expression of any gene under 5 different promoters: ubiquitous (*eft-3p* -*eef-1A.1p*-), neuronal (*rab-3p*), muscle (*myo-3p*), germline (*pie-1p*), and intestinal (*elt-2p*) promoters (Silva-García et al. 2019). Taking advantage of these well-characterized harbor loci, we inserted the SKI PLACE cassettes in these regions for chromosomes I, III, IV, and V. For chromosomes II and X, we identified new intergenic sites (see Materials and Methods section for details). All insertions were carried out using the cloning-free and in vitro assembly CRISPR/Cas9 approach (Paix et al. 2015, 2016; Silva-García et al. 2019).

We generated 6 transgenic strains with an equal cassette design. Each strain consists of the 2 exact 900-bp HAs present in the pSKI plasmid (Fig. 1a and b), and between them is a CRISPR target sequence copied from the *dpy-10* gene: HA1::*dpy-10* site::HA2 (Fig. 1b). By inserting a 30-base protospacer and PAM sequence from *dpy-10* gene (Arribere et al. 2014), hereafter referred to as “*dpy-10* site,” we can simultaneously induce double-stranded breaks at both (i) the cassette *dpy-10* site and (ii) the endogenous *dpy-10* locus using a single crRNA guide. Other studies, including our own, have demonstrated that this approach is cost-effective, requiring only 1 crRNA guide (Mouridi et al. 2017; Silva-García et al. 2019). In order to introduce the *dpy-10* site into the cassette, we used another well-established and identifiable Co-CRISPR target gene, *dpy-5* (Silva-García et al. 2019). We have generated 6 strains, with the cassette introduced into defined intergenic regions in all chromosomes (Fig. 1c). We engineered all strains following the CRISPR/Cas9 protocol based on in vitro assembly and using PCR products as templates (Paix et al. 2015, 2016; Silva-García et al. 2019). We did 1 CRISPR edit step to introduce the final cassette using 2 overlapping PCR fragments (see Materials and methods). Finally, all SKI PLACE end lines were outcrossed at least 6 times to eliminate potential off-targets. After outcrossing, we analyzed the strains for fertility, generation time, and lifespan (Fig. 1, d to f). Although the SKI PLACE strains do not show dramatic defects, we still observed some minor significant differences in generation time and lifespan (Fig. 1, e to f).

### Testing the SKI PLACE system

To verify that our SKI PLACE system could be used to introduce a transgene in 1 step to drive single-copy expression, we (i) cloned a fluorescent reporter gene into the pSKI plasmid and (ii) inserted the reporter gene into all outcrossed SKI PLACE strains by CRISPR knock-in, using the pSKI as a repair template.

**Fig. 2.**
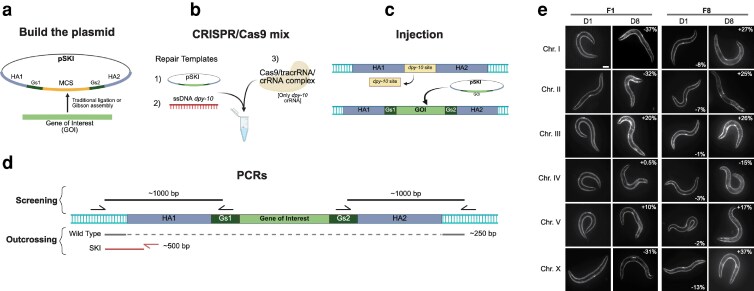
Workflow for using the SKI PLACE system to generate single-copy insertions. a) The gene of interest (GOI) is cloned into the pSKI plasmid. b) Assemble CRISPR/Cas9 mix in vitro using the pSKI plasmid as a repair template. c) Injection of this mix replaces the *dpy-10* site with the GOI. d) Using the SKI primers, the insertion of the GOI is screened either in the 5′ region (genotyping sequence 1, Gs1) or the 3′ region (genotyping sequence 2, Gs2). After identifying and confirming the GOI integration, another set of primers is used to outcross the SKI strain. For details on cloning, insertion, and screening, refer to the step-by-step user guide in Supplementary File 1. e) SKI PLACE strains were tested by knocking in the mCherry fluorescent protein, driven by the *myo-3* promoter (see details in the text). D, day; F, generation. The percentage at the top of each image in D8 animals represents the comparison of fluorescent intensity between older and younger animals in both generations (D1 vs D8), and the percentage at the bottom of F8-D1 animals indicates the comparison between generations (F1-D1 vs F8-D1).

Using the Gibson assembly protocol (Gibson et al. 2009), we cloned the following transgene into the pSKI plasmid: *myo-3p::mCherry::unc-54 3′ UTR* (see Materials and methods) (Fig. 2a). Without including the HAs, the total length of this fluorescent reporter is 4091 bp and drives mCherry expression in wall muscles. CRISPR/Cas9 mix was assembled in vitro (Silva-García et al. 2019) using this pSKI-reporter plasmid as a repair template (Fig. 2b). As expected, utilizing only 1 crRNA guide, we obtained *dpy-10* mutants (Co-CRISPR marker) that also showed muscular expression of mCherry in all our SKI PLACE strains. Because not all GOI carry a fluorescent reporter to track, or the fluorescence is not strong enough to be detected under a standard dissecting UV microscope, we specifically designed our pSKI plasmid with 2 sequences (Genotyping sequence 1 and 2, Gs1 and GS2) for screening purposes: HA1::Gs1::GOI::Gs2::HA2 (Fig. 2a). These Gs1 and Gs2 sequences are always introduced along with the GOI and serve as genotyping sequences for both 5′ and 3′ ends (Fig. 2c). Each Gs1 and Gs2 region amplifies a fragment around 1000 bp, which is used to screen dumpy animals for candidates with potential single-copy insertions by PCR (Fig. 2d, screening). We have designed and tested primers that can be used to screen the strains (see the step-by-step guide in Supplementary File 1). We have also designed and tested primers to outcross the final SKI PLACE lines that carry the GOI into the N2 wild type (Fig. 2d, outcrossing and Supplementary File 1).

We used the PCR strategy for the Gs1 and Gs2 regions to identify candidates with potential single-copy insertions of the *myo-3p::mCherry::unc-54 3′ UTR* (Fig. 2d, screening). We analyzed the HA1 5′ and HA2 3′ ends by PCR, with a final percentage of animals showing both PCR hits of 3.1%, 7.5%, 4.7%, 6.6%, 23.5%, and 6.2% for chromosomes I, II, III, IV, V, and X, respectively (Table 2). We also evaluated the number of imprecise insertions (Tables 2 and Supplementary 3). We categorize an imprecise insertion into 2 classes. (i) When the PCR in each arm does not show the correct expected band. (ii) When the PCR shows the correct band but only in 1 arm, it suggests partial insertions. The SKI PLACE strains exhibit an imprecise insertion percentage of class 1, ranging from 0 to 4.4%, with the HA1 in chromosome I being the highest. Notably, chromosomes V and X do not display different PCR sizes in our hands (Table 2). For the class 2 of potential partial insertions, the HA1 shows a higher number of correct PCR bands only in this arm for chromosome II (21.9%) compared to the HA2 region in the same chromosome (1.4%) (Supplementary Table 3). Our data suggests that the HA1 region is more prone to imprecise insertions or deletions. Then, we selected at least 1 positive transgenic strain from each chromosome to analyze mCherry expression. The SKI PLACE transgenic lines were outcrossed and maintained for at least 8 generations. Although we observed that these strains maintain relative transgene expression across generations, they show a decrease in transgene expression with age, with the SKI PLACE strain of chromosome I showing the highest reduction at day 8 (−37%) (Fig. 2e).

**Table 2.** Homology-directed repair (HDR) efficiencies in the SKI PLACE strains.

Strain (Chr.)	dpy/rol F1 screened^a^	% (*n*) both HA precise	% (*n*) HA1 precise	% (*n*) HA1 imprecise	% (*n*) HA2 precise	% (*n*) HA2 imprecise
**Chr. I** CSG18	160	3.1 (5)	8.1 (13)	4.4 (7)	11.3 (18)	0.6 (1)
**Chr. II** CSG60	146	7.5 (11)	29.5 (43)	0.7 (1)	8.9 (13)	2.1 (3)
**Chr. III** CSG36	234	4.7 (11)	12.0 (28)	0.4 (1)	6.4 (15)	0.9 (2)
**Chr. IV** CSG10	182	6.6 (12)	11.5 (21)	0.5 (1)	8.8 (16)	2.2 (4)
**Chr. V** CSG76	115	23.5 (27)	35.7 (41)	0 (0)	28.7 (33)	0 (0)
**Chr. X** CSG53	146	6.2 (9)	8.2 (12)	0 (0)	11.6 (17)	0 (0)

^a^Fourteen worms were injected for each strain.

Overall, our SKI PLACE system allows single-copy insertions. The desired transgene can be cloned into the pSKI plasmid, and, as an added value, it can also be shared and edited among the *C. elegans* community. The SKI PLACE strains offer the option to select the target chromosome to insert the GOI. Finally, the efficiency of CRISPR editing depends on several factors (some of them discussed here (Farboud et al. 2019; Ghanta et al. 2021). In the case of our system, we have found that achieving a successful insertion depends on the purity of the plasmid, the CRISPR mix, a well-honed microinjection technique, and a clean PCR screening.

## Discussion

Here, we present the SKI PLACE toolkit for generating single-copy insertions into the *C. elegans* genome at specific harbor locations. We engineered the pSKI plasmid to function as a repair template for CRISPR/Cas9-based insertions. We also developed a set of SKI PLACE strains that enable the insertion of the transgene from the pSKI plasmid.

What does our SKI PLACE system contribute to the existing CRISPR-based options for expressing single-copy transgenes in *C. elegans*? Several techniques have been developed to express single-copy transgenes in the nematode genome. Among the most recent transgenic methods are the use of Flp, Cre, and phiC31 recombinases (Nonet 2020, 2023; Yang et al. 2022), Split-wrmScarlet and split-sfGFP (Goudeau et al. 2021), modular safe-harbor transgene insertion (MosTI) (Mouridi et al. 2022), and the Mos1-mediated Single-Copy Insertion (MosSCI) method, which is widely used to establish stable transgenic strains (Frøkjær-Jensen et al. 2008, 2012, 2014; Fan et al. 2020). All these methods have been beautifully designed and have positively advanced the generation of single-copy transgenic lines. However, *C. elegans* researchers usually decide whether to use efficient methods to integrate single-copy transgenes at random sites or less efficient and laborious methods to target a specific genomic site. Thus, our toolkit complements current transgenic methods, offering some advantages. We have specifically developed the pSKI plasmid with a multi-cloning site sequence (MCS) (Fig. 1a). Our SKI PLACE strains are manageable to inject and are designed to avoid disruptions in *C. elegans* genes, as we have synthetically designed the homology arms (HAs) and integrated them into specific intergenic regions. Having the cassette in all chromosomes gives the choice to select the desired targeted region (Fig. 1b). The SKI PLACE primers that target the genotyping sequences (Gs1 and Gs2) streamline the PCR screening, although the researcher needs to corroborate the entire insertion at the end by sequencing. The pSKI plasmid can also be used for extrachromosomal arrays. In another scenario, suppose a researcher has already cloned their gene of interest (GOI) into a plasmid for extrachromosomal expression; in this case, digestion and ligation can be used to extract the GOI and introduce it into the pSKI plasmid. Lastly, although selection markers are a necessary tool in *C. elegans* genetics to identify successfully transformed animals, they can introduce unintended side effects, especially when these markers cannot be removed by outcrossing. Moreover, these markers require the use of specific mutant animals as recipient strains, which are generally more difficult to grow than the wild type, may complicate genetic interaction studies, and influence phenotype, physiology, and gene expression (Giordano-Santini and Dupuy 2011; Cornes et al. 2014). Thus, our SKI PLACE system provides the option to remove the selection marker by outcrossing the strains. Table 3 lists some advantages and potential combinations that can be applied along with our system.

**Table 3.** Advantages and potential combinations for the SKI PLACE system.

47 single restriction cuts in the pSKI plasmid to clone the gene of interest
Use the pSKI plasmid as extrachromosomal array
Instead of the pSKI plasmid, use PCR templates or ssODNs (Paix et al. 2017) that have homology arms that match the homology arms HA1 and HA2 in the SKI PLACE strains
Introduce the gene of interest into the pSKI plasmid that has already been cloned into another plasmid
Use the pSKI plasmid for plasmid-based CRISPR mix (Kim and Colaiácovo 2019) instead of ribonucleoprotein assembly
The pSKI plasmid is easy to edit and share within *C. elegans* community
Co-inject the *peel-1* negative selection marker to kill animals harboring transgenes as extrachromosomal arrays (McDiarmid et al. 2020)
Cross the SKI PLACE lines into the Cas9 integrated strain (Schwartz et al. 2021)
Add an antibiotic resistance gene (hygromycin [Stevenson et al. 2020]) separated from the gene of interest by the trans-splicing sequence (Blumenthal 2012) into the pSKI plasmid for selection
There is no need to design primers for screening or outcrossing

Although the cassette is inserted into all chromosomes, more strains are needed in order to provide at least 2 options for each chromosome. Screening multiple worms by PCR is also a limitation in our system. Since this single-copy approach is based on the CRISPR/Cas9 technique, our system is more suitable for researchers who are confident in their ability to work with this technique. Large insertions are always a challenge, and the efficiency of insertion depends on several factors. For example, in our hands, we have observed that a successful CRISPR edit relies on several factors, including transgene length, sequence complexity, mix purity, homology arms, and microinjection proficiency. In addition, the transgene structure can significantly impact its expression. For instance, we have previously demonstrated that introns and 3′ UTRs are crucial for ensuring proper transgene expression, particularly in the germline (Silva-García et al. 2019). Thus, taking into consideration these points and the available single-copy knock-in approaches, we believe that the decision on which system to use to generate single-copy transgenes will ultimately depend on the researcher's experience.

Finally, all SKI PLACE reagents are available freely to the *C. elegans* community. A step-by-step user guide is included in Supplementary File 1. Strains reported here, new strains, updated protocols, and all sequences can be found at https://www.theSGlab.com/resources.

## Supplementary Material

jkaf220_Supplementary_Data

## Data Availability

All reagents, including strains, plasmids, and all derived sequences from sequencing, are available upon request. The authors affirm that all data necessary to confirm the conclusions of the article are presented within the manuscript, figures, and tables. Supplementary material, including all sequence files for the SKI PLACE lines, is available at FigShare (https://doi.org/10.25387/g3.29743289). Supplemental material available at G3 online.
