# Supplementary material for: A single-copy knock-in system: one plasmid to target all chromosomes in C. elegans
Source: G3 (Bethesda). 2025 Sep 19;15(11):jkaf220. doi: 10.1093/g3journal/jkaf220 (PMC12608071; doi:10.1093/g3journal/jkaf220)
Supplement: jkaf220_Supplementary_Data [file jkaf220_supplementary_data.zip › File_S1_Step_by_step_guide_G3-2025-406217.pdf]

Step-by-step guide for using the SKI PLACE System

Strains reported here, new strains, updated protocols, and all sequences can be found at
https://www.theSGlab.com/resources

Step 1

Clone your transgene of interest (GOI) in the pSKI plasmid by traditional cloning or Gibson reaction.

- HA: Homology arms (1 and 2); 900 bp each.
- Gs: Genotyping sequence (1 and 2); 50 bp each.
- MCS: Multi-cloning site sequence (47 cuts); 222 bp.
- AscI and FseI restriction sites (less likely to be found in the *C. elegans* genome).
- pSKI: 4728 bp and Amp resistant.
- The pSKI plasmid can be used for extrachromosomal arrays.

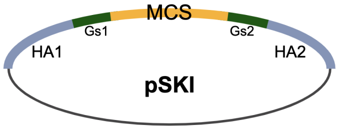

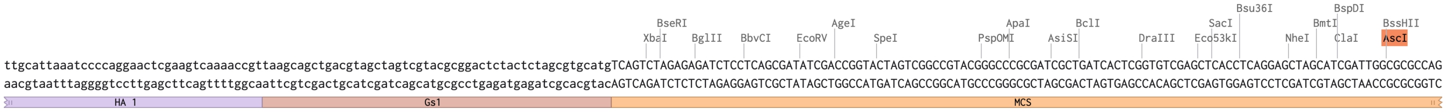

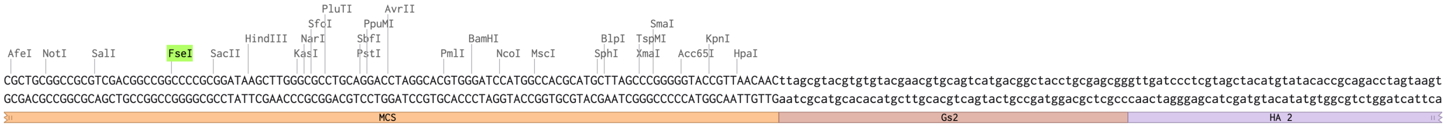

Step 2

Select the SKI PLACE strain to knock in your gene of interest.

- All strains have the following cassette:

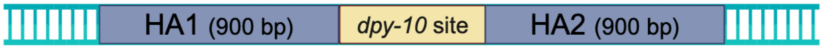

- Strains and chromosome positions:

Chromosome	Position	Strain
I	2,850,968	CSG18
II	9,834,540	CSG60
III	7,007,779	CSG36
IV	5,014,948	CSG10
V	8,644,845	CSG76
X	798,667	CSG53

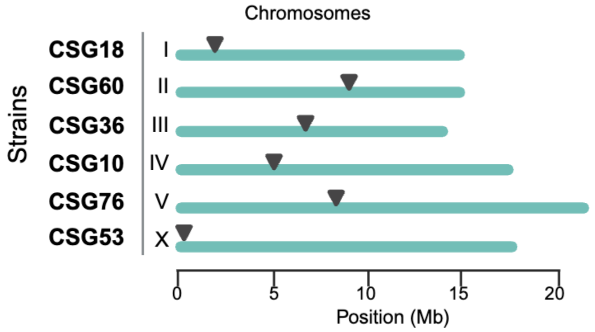

Step 3

Assemble of CRISPR/Cas9 complex *in vitro*:

	Reagent	Volume (µl)
**1**	H_2O	Up to 10
**2**	Hepes pH 7.4 (200 mM)	0.375
**3**	KCl (1 M)	0.25
**4**	tracrRNA (4 µg/µl)	2.5
**5**	*dpy-10* crRNA (2.6 µg/µl)	0.6
**6**	*dpy-10* ssODN (500 ng/µl)	0.25
**7**	***pSKI plasmid Template (ng/µl)***	*
**8**	Purified Cas9 (12 µg/µl)	2.0
	**Total:**	**10 µl**

*Note that we have had great success getting CRISPR edits using a final concentration of pSKI template of ~200 ng/µl in the CRISPR mix.

- Injection mixes can be prepared in advance without Cas9 protein, separated into 2 tubes of 4 µl, and stored at -80ºC.
- We have found that frozen mixes stored for up to one year at -80ºC are still effective at generating CRISPR edits.
- Before injecting, thaw one 4 µl mix, add 1 µl of purified Cas9 (12 µg/µl), mix by pipetting, spin for 2 min at 13000 rpm, and incubate at 37 ºC for 10 min.
- Inject into day 1 adults of the relevant SKI PLACE strain.

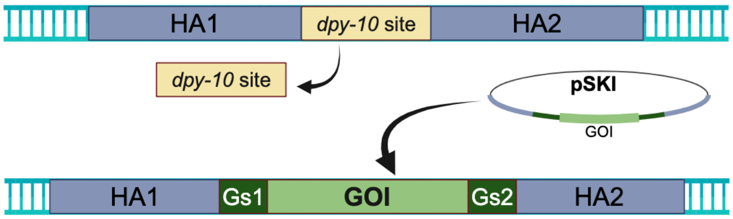

Step 4

3-4 days after injection, screen for plates that have produced many dumpy and/or roller progenies.

- From these plates, individually plate single *dpy* or *rol* animals and allow them to lay eggs before screening them for your desired edit. We suggest screening at least 100 worms; we usually screen between 100-150 *dpy*/*rol* animals.
- Screening can be performed by examining fluorescent protein expression and/or by genotyping them using PCR.
- We have designed two specific sequences (Gs1 and Gs2) for PCR genotyping.
- We have also annotated and tested additional SKI Primers to facilitate the screening process.
- The screening consists of two independent PCRs:

1. PCR 1: Amplify the 5’ region using the Gs1 sequence.
2. PCR 2: Amplify the 3’ region using the Gs2 sequence.

- **Note**: If you design new primers for genotyping, avoid designing them within the HA1 or HA2 regions, as these homology arm sequences are also present in the pSKI plasmid. Then, design primers inside your gene of interest.
- Use the following model, table, and gels as a reference for PCR screening.

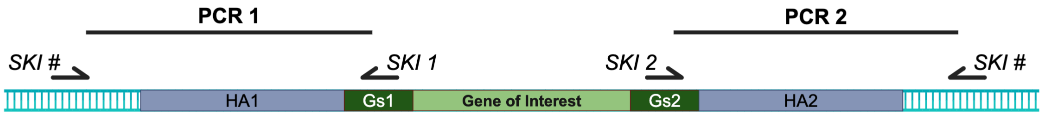

PCR 1				
**Strain**	**SKI # primer FOR (genomic)**	**SKI 1 primer REV (Gs1)**	**Band (bp)**	Example
**Chr. I CSG18**	SKI 4<br>5’ tcacaccttttctctcgtctctcc	SKI 1<br>5’ tgcacgctagagtagagtccg	1033	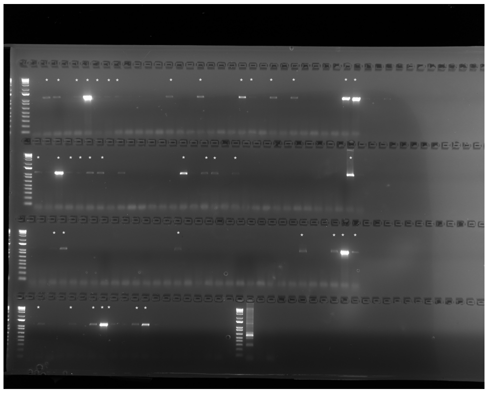
**Chr. II CSG60**	SKI 5<br>5’ gtcttgctaccgatcaacccac		1047	
**Chr. III CSG36**	SKI 6<br>5’ cctgggaacaataagtcggtgaag		1167	
**Chr. IV CSG10**	SKI 7<br>5’ gaactttgcagtttggttgtagtg		1099	
**Chr. V CSG76**	SKI 8<br>5’ accttcgacctcactttccctc		1068	
**Chr. X CSG53**	SKI 14<br>5’ agttgtcggattgctcacttgg		1153	

PCR 2				
**Strain**	**SKI 2 primer FOR (Gs2)**	**SKI # primer REV (genomic)**	**Band (bp)**	Example
**Chr. I CSG18**	SKI 2<br>5’ agcgtacgtgtgtacgaacg	SKI 9<br>5’ cctcccctcatctcaattatcccg	1097	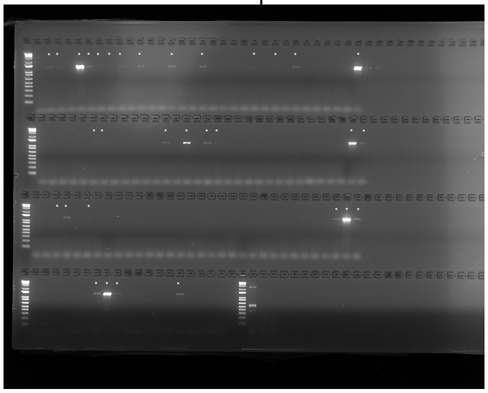
**Chr. II CSG60**		SKI 10<br>5’ gtttttggttatcgcggcacag	1088	
**Chr. III CSG36**		SKI 11<br>5’ aatgtctggcggtccaaagtg	1171	
**Chr. IV CSG10**		SKI 12<br>5’ tcaatccgttcatttgagccc	1049	
**Chr. V CSG76**		SKI 13<br>5’ ccgtcctgaagtatacccagatcc	1185	
**Chr. X CSG53**		SKI 15<br>5’ cactgtcgcttatttggacccc	1180	

- PCR conditions:

PCR mix		PCR cycle		
**Reagent**	**Volume (µl)**	**Steps**	**Conditions**	**Repeats**
H_2O	8.8	**1**	95º 5’	
SKI FOR (100 µM)	0.1	**2**	95º 30’’	
SKI REV (100 µM)	0.1	**3**	**57º** 30’’	33-35x (Steps 2-4)
Red Apex 2x[#]	10	**4**	72º 1’30’’	
Worm lysis	1	**5**	72º 5’	
TOTAL	**20 µl**	**6**	12º α	

[#]Genesee Scientific, it contains Apex Taq DNA polymerase, an ammonium-based buffer system, Apex ultra-pure dNTPs (>99%), magnesium chloride, and a Red Dye for visualization.

- **Note**: In our PCR gel examples, there are hits that show bright and dim bands. We do not have a preference for how these bands appear, as this could be a direct result of the PCR and gel processes, rather than the integration. We recommend selecting any animal/plate that shows positive results for both HA, including those that are bright, dim, or a combination of the two.

Step 5

From those F1 animals that screen positive for **both** PCR1 (5’ region) and PCR2 (3’ region), go back to the plates they were picked from and pick 4-6 of their F2 progeny to individual plates, allow them to lay eggs, and then screen them as above. Continue doing this until getting homozygous animals for your desired edit. Confirm that the knock-in CRISPR edit is correct by sequencing.

Step 6

Outcross your newly made SKI PLACE line to eliminate the *dpy-10* Co-CRISPR edit.

- Note that the outcrossing will use the wild-type N2 strain, not the SKI PLACE recipient strains.
- The following model and table show primers flanking the cassette that can be used to outcross the new SKI PLACE line to N2.
- Note that the external primers used for the CRISPR screening are the same as those used here.
- Follow the same PCR conditions described above, but reduce the extension time to 1 minute.

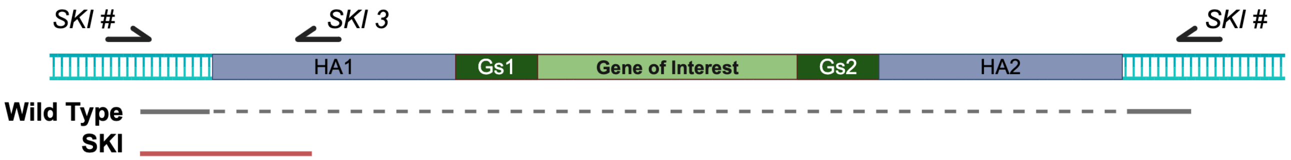

Outcrossing: 3 primers strategy					
**Strain**	**SKI primer FOR (genomic)**	**SKI 3 primer (HA1)**	**SKI primer REV (genomic)**	**Wild-type band (bp)**	**SKI band (bp)**
**Chr. I CSG18**	SKI 4<br>5’ tcacaccttttctctcgtctctcc	SKI 3<br>5’ ttacggtgtaggagacagggtc	SKI 9<br>5’ cctcccctcatctcaattatcccg	234	545
**Chr. II CSG60**	SKI 5<br>5’ gtcttgctaccgatcaacccac		SKI 10<br>5’ gtttttggttatcgcggcacag	239	559
**Chr. III CSG36**	SKI 6<br>5’ cctgggaacaataagtcggtgaag		SKI 11<br>5’ aatgtctggcggtccaaagtg	442	679
**Chr. IV CSG10**	SKI 7<br>5’ gaactttgcagtttggttgtagtg		SKI 12<br>5’ tcaatccgttcatttgagccc	252	611
**Chr. V CSG76**	SKI 8<br>5’ accttcgacctcactttccctc		SKI 13<br>5’ ccgtcctgaagtatacccagatcc	357	580
**Chr. X CSG53**	SKI 14<br>5’ agttgtcggattgctcacttgg		SKI 15<br>5’ cactgtcgcttatttggacccc	437	665

Step 7 *(optional)*

The major predicted off-target site of the *dpy-10* crRNA is in an exon of R12E2.15. After outcrossing, to check potential off-target events from the *dpy-10* crRNA, we recommend using primers FOR (5’ gttgggtatgctcctccttgtg) and REV (5’ agaagactacatacgacggctgg). These primers amplify a fragment of 873 bp from the R12E2.15 gene, flanking the potential off-target sequence. Use this PCR product for sequencing with the same primers.
