# Supplementary material for: A single-copy knock-in system: one plasmid to target all chromosomes in C. elegans
Source: G3 (Bethesda). 2025 Sep 19;15(11):jkaf220. doi: 10.1093/g3journal/jkaf220 (PMC12608071; doi:10.1093/g3journal/jkaf220)
Supplement: jkaf220_Supplementary_Data [file jkaf220_supplementary_data.zip › Table_S1_G3-2025-406217.pdf]

Table S1. crRNAs and tracrRNA used in this study

crRNA Name	Target gene/region	Sequence (5’>3’)
dpy-10 crRNA (Arribere et al. 2014)	*dpy-10*	gctaccataggcaccacgag
dpy-5 crRNA (Silva-García et al. 2019)	*dpy-5*	ccaggaatgccaggaccacg
ttTi4348 crRNA Ch. I	I:2850968	ttttgtcaaaagaagagaca
oxTi179 crRNA Chr. II	II:9834540	gatcaacgaaaaaagatatg
oxTi444 crRNA Chr. III	III:7007779	ggtatatagttaaataaacg
cxTi10816 crRNA Chr. IV	IV:5014948	acaagtgtgaaactaaactt
oxTi365 crRNA Chr. V	V:8644845	aacaagttgggacaatacgg
crRNA Chr. X	X:798667	aagatggggtaccatctgtg
tracrRNA	N/A	aacagcauagcaaguuaaaauaaggcuaguccguau caacuugaaaaaguggcaccgagucggugcuuuu

References

Arribere JA et al. 2014. Efficient Marker-Free Recovery of Custom Genetic Modifications with CRISPR/Cas9 in Caenorhabditis elegans. Genetics. 198(3):genetics.114.169730. https://doi.org/10.1534/genetics.114.169730

Silva-García CG et al. 2019. Single-Copy Knock-In Loci for Defined Gene Expression in Caenorhabditis elegans. G3: GenesGenomesGenet. 9(7):2195–2198. https://doi.org/10.1534/g3.119.400314
