# Supplementary material for: A single-copy knock-in system: one plasmid to target all chromosomes in C. elegans
Source: G3 (Bethesda). 2025 Sep 19;15(11):jkaf220. doi: 10.1093/g3journal/jkaf220 (PMC12608071; doi:10.1093/g3journal/jkaf220)
Supplement: jkaf220_Supplementary_Data [file jkaf220_supplementary_data.zip › Table_S2_G3-2025-406217.pdf]

Table S2. Oligonucleotides used in this study.

Oligo Name	Sequence 5’ > 3’	Template	SKI PLACE Strain or Plasmid
GS3	acagttcatcgactagtgtgagacaagtgtgaaactaaatgttgctatc cacgtaacaac	HA1	CSG10
GS4	gatcaacgctccgctaccataggcaccacgagcggtaacggttttga cttcgagttcc	HA1	CSG10, CSG18, CSG60, CSG76, CSG36, CSG53
GS5	aaaaccgttaccgctcgtggtgcctatggtagcggagcgttgatccctc gtagctacatg	HA2	CSG10, CSG18, CSG60, CSG76, CSG36, CSG53
GS6	ctcctttaagttacgattattaacaaaatgtcgcctaaggaaattaaggc ctccaatgtg	HA2	CSG10
GS1 (SKI 7)	gaactttgcagtttggttgtagtg	Genotyping	CSG10
GS7	tcagtgtagcccaccttctgtaag	Genotyping	CSG10, CSG18, CSG60, CSG76, CSG36, CSG53
GS8	acgataatggtcgtgcgatgtc	Genotyping	CSG10, CSG18, CSG60, CSG76, CSG36, CSG53
GS2 (SKI 12)	tcaatccgttcatttgagccc	Genotyping	CSG10
ED14	tgaatctcactctgatgagcgtatctatcaagtccttgttgttgctatccac gtaacaac	HA1	CSG18
ED15	caagtcggcgatttctttgaagttttgtcaaaagaagaggaaattaagg cctccaatgtg	HA2	CSG18
ED16 (SKI 4)	tcacaccttttctctcgtctctcc	Genotyping	CSG18
ED17 (SKI 9)	cctcccctcatctcaattatcccg	Genotyping	CSG18
ED6 (SKI 3)	ttacggtgtaggagacagggtc	Genotyping	CSG10, CSG18, CSG60, CSG76, CSG36, CSG53
ED18	ggagagcccctagtaaaacattgatcaacgaaaaaagattgttgctat ccacgtaacaac	HA1	CSG60
ED19	acatgagaaagagcacagagcaaaaacaattttccccatgaaatta aggcctccaatgtg	HA2	CSG60
ED20 (SKI 5)	gtcttgctaccgatcaacccac	Genotyping	CSG60
ED21 (SKI 10)	gtttttggttatcgcggcacag	Genotyping	CSG60
ED22	ctttttgagttaaataaatgaggtatatagttaaataatgttgctatccac gtaacaac	HA1	CSG36
ED23	atgctctccagttgaagatctgaagcccatatacctcgtgaaattaagg cctccaatgtg	HA2	CSG36
ED24 (SKI 6)	cctgggaacaataagtcggtgaag	Genotyping	CSG36
ED25 (SKI 11)	aatgtctggcggtccaaagtg	Genotyping	CSG36
ED26	atcactaatgtcaccgtttcgtcgcgtgtcgctcccccgtgttgctatcca cgtaacaac	HA1	CSG76
ED27	gaaatggagaaggaccgagtagaacaagttgggacaatagaaatt aaggcctccaatgtg	HA2	CSG76
ED28	accttcgacctcactttccctc	Genotyping	CSG76

(SKI 8)			
ED29 (SKI 13)	ccgtcctgaagtatacccagatcc	Genotyping	CSG76
ED48 (SKI 1)	tgcacgctagagtagagtccg	Genotyping	CSG10, CSG18, CSG60, CSG76, CSG36, CSG53
ED49 (SKI 2)	agcgtacgtgtgtacgaacg	Genotyping	CSG10, CSG18, CSG60, CSG76, CSG36, CSG53
ED35	gaactcgaagtcaaaaccgttaagcagctgacgtagctagtcgtacg cggactctactctagcgtgcatgtcagtctagagagatctcctc	Gs1	pSKI
ED36	tgtagctacgagggatcaacccgctcgcaggtagccgtcatgactgc acgttcgtacacacgtacgctaagttgttaacggtacccccgg	Gs2	pSKI
ED7	gctaacaacttggaaatgaaatatgttgctatccacgtaacaacaaat ag	HA1	pSKI
ED8	gggcccgtacggccgactagtaccggtcgatatcgctgaggag	HA1	pSKI
ED9	accggtactagtcggccgtacgggcccgcgatcgctgatcactcggt g	HA2	pSKI
ED10	caccgaaacgcgcgagacgaaaggaaattaaggcctccaatgtgc	HA2	pSKI
ED43	ggactctactctagcgtgcatgtcagtagtgattatagtctctgttttcg	*myo-3p*	pED4
ED1	ttgaattgtattgagccttcggagcg	*myo-3p*	pED4
ED2	caactcgctccgaaggctcaatac	*mCherry::3' UTR*	pED4
ED44	acacacgtacgctaagttgttaacgcttccactgagcctcaaacccaa ac	*mCherry::3' UTR*	pED4
ED57	ttggtaaatttgtgcagtctttaagatggggtaccatcttgttgctatccac gtaacaac	HA1	CSG53
ED58	ttttagcactatacgagtggtttttaatcatctccccacgaaattaaggcc tccaatgtg	HA2	CSG53
ED59 (SKI 14)	agttgtcggattgctcacttgg	Genotyping	CSG53
ED60 (SKI 15)	cactgtcgcttatttggacccc	Genotyping	CSG53
