# Supplementary material for: A single-copy knock-in system: one plasmid to target all chromosomes in C. elegans
Source: G3 (Bethesda). 2025 Sep 19;15(11):jkaf220. doi: 10.1093/g3journal/jkaf220 (PMC12608071; doi:10.1093/g3journal/jkaf220)
Supplement: jkaf220_Supplementary_Data [file jkaf220_supplementary_data.zip › Table_S3_G3-2025-406217.pdf]

Table S3. Potential partial insertions in the SKI PLACE strains.

Strain (Chr.)	**dpy F1 screened**	**% (n) HA1 partial insertions**	**% (n) HA2 partial insertions**
**Chr. I** CSG18	160	5.0 (8)	8.1 (13)
**Chr. II** CSG60	146	21.9 (32)	1.4 (2)
**Chr. III** CSG36	234	7.3 (17)	1.7 (4)
**Chr. IV** CSG10	182	4.9 (9)	2.2 (4)
**Chr. V** CSG76	115	12.2 (14)	5.2 (6)
**Chr. X** CSG53	146	2.1 (3)	5.5 (8)
