# Supplementary material for: A single-copy knock-in system: one plasmid to target all chromosomes in C. elegans
Source: G3 (Bethesda). 2025 Sep 19;15(11):jkaf220. doi: 10.1093/g3journal/jkaf220 (PMC12608071; doi:10.1093/g3journal/jkaf220)
Supplement: jkaf220_Supplementary_Data [file jkaf220_supplementary_data.zip › Table_S4_G3-2025-406217.pdf]

**Table S4. Strains generated to verify the SKI PLACE lines.**

| Chromosome | Strain | Genotype | crRNA used | Recipient Strain | Outcrossed |
|---|---|---|---|---|---|
| Chr. I | CSG78 | *CSG18, gsgIs13 [synthetic 900bp HA left::myo-3p::mCherry::unc-54 3'UTR:: synthetic 900bp HA right I: 2850968]* | *dpy-10 crRNA* | CSG18 | 2x |
| Chr. II | CSG85 | *CSG60, gsgIs10 [synthetic 900bp HA left::myo-3p::mCherry::unc-54 3'UTR:: synthetic 900bp HA right II: 9834540]* | *dpy-10 crRNA* | CSG60 | 2x |
| Chr. III | CSG69 | *CSG36, gsgIs7 [synthetic 900bp HA left::myo-3p::mCherry::unc-54 3'UTR:: synthetic 900bp HA right III: 7007779]* | *dpy-10 crRNA* | CSG36 | 4x |
| Chr. IV | CSG70 | *CSG10, gsgIs9 [synthetic 900bp HA left::myo-3p::mCherry::unc-54 3'UTR:: synthetic 900bp HA right IV: 5014948]* | *dpy-10 crRNA* | CSG10 | 2x |
| Chr. V | CSG68 | *CSG76, gsgIs6 [synthetic 900bp HA left::myo-3p::mCherry::unc-54 3'UTR:: synthetic 900bp HA right V:8644845]* | *dpy-10 crRNA* | CSG76 | 4x |
| Chr. X | CSG86 | *CSG53, gsgIs11 [synthetic 900bp HA left::myo-3p::mCherry::unc-54 3'UTR:: synthetic 900bp HA right X: 798667]* | *dpy-10 crRNA* | CSG53 | 2x |
